# Dietary behaviour, psychological well-being and mental distress among adolescents in Korea

**DOI:** 10.1186/s13034-017-0194-z

**Published:** 2017-11-28

**Authors:** Seo Ah Hong, Karl Peltzer

**Affiliations:** 1 ASEAN Institute for Health Development, Mahidol University, Salaya, Phutthamonthon, Nakhon Pathom 73170 Thailand; 2 Institute for Health and Society, Hanyang University, Seoul, Republic of Korea; 3 Department for Management of Science and Technology Development, Ton Duc Thang University, Ho Chi Minh City, Vietnam; 4 Ton Duc Thang University, Ho Chi Minh City, Vietnam

## Abstract

**Background:**

Dietary intake is important for physical and mental health. The aim of this investigation was to assess associations between dietary behaviours and psychological well-being and distress among school-going adolescents in Korea.

**Methods:**

In a cross-sectional nationally representative survey, 65,212 students (Mean age = 15.1 years, SE = 0.02 and 52.2% male and 47.8% female) responded to a questionnaire that included measures of dietary behaviour, psychological well-being and mental distress.

**Results:**

In logistic regression analyses, adjusted for age, sex, socioeconomic status, school level, school types, Body Mass Index, physical activity, and substance use, positive dietary behaviours (regular breakfast, fruit, vegetable, and milk consumption) were positively and unhealthy dietary behaviours (intake of caffeine, soft drinks, sweet drinks and fast food consumption) were negatively associated with self-reported health, happiness and sleep satisfaction. Positive dietary behaviours (regular breakfast, fruit, vegetable, and milk consumption) were negatively associated with perceived stress and depression symptoms. Unhealthy dietary behaviours (consumption of fast food, caffeine, sweetened drinks and soft drinks) were associated with perceived stress and depression symptoms.

**Conclusions:**

The study found strong cross-sectional evidence that healthy dietary behaviours were associated with lower mental distress and higher psychological well-being. It remains unclear, if a healthier dietary behaviour is the cause or the sequela of a more positive well-being.

## Background

Recently, more studies have been trying to link dietary behaviour to psychological well-being and distress [1–6]. Regular fruit, vegetable and breakfast intake (healthy dietary behaviours) have been found positively associated with self-reported health, happiness, and better sleep [1–8], and regular fruit, vegetable and breakfast intake were negatively associated with perceived stress, mental distress and depression [1–3, 9–25]. Further, specific unhealthy dietary behaviours (consumption of soft drinks, fast food, sweets and snacks, skipping breakfast, and caffeine) were associated with unhappiness, perceived stress, mental or psychological distress, depression or poorer sleep [5, 8, 19, 24–36]. Mixed results were found in relation to the consumption of milk and psychological well-being. One study found that increased milk product consumption was associated with depression [37], Meyer et al. [38] found milk consumption improves sleep quality, and Aizawa et al. [39] found that the frequency of fermented milk consumption was associated with higher Bifidobacterium counts and that patient with major depressive disorder have lower Bifidobacterium and/or Lactobacillus counts.

In a study among Iranian children and adolescents junk food consumption (such as fast foods, sweets, sweetened beverages, and salty snacks) was significantly associated with mental distress, including “worry, depression, confusion, insomnia, anxiety, aggression, and feelings of being worthless.” [26] Fast food consumption was associated with depression among adolescent girls in Korea [32], and among Chinese adolescents, snack consumption was associated with psychological symptoms [34]. The poor nutrient content of junk or fast foods may have an effect on normal brain functioning and, thus, have an effect on negative mood via the synthesis of neurotransmitters such as serotonin [40, 41]. In a study among adolescents in Norway, a J-shaped relationship between soft drink consumption and mental distress was found [42]. The effects of soft drink or sugar consumption on mental health may be mediated through other nutritional or behavioural factors [42]. Among secondary school students in Malaysia, regular breakfast consumption was negatively associated with mild or moderate stress [23]. In a large study of adolescent school-going children (N = 3071) from the United Kingdom, positive relationships between caffeine consumption and anxiety and depression were found [33]. It is possible that students used caffeinated products to cope with stress [33, 43].

We have limited information on the relationship between dietary behaviour, psychological well-being and mental distress among adolescents in Asia, which prompted this study. It was hypothesized that healthy dietary behaviour enhances psychological well-being and reduces mental distress, and unhealthy dietary behaviours reduce psychological well-being and increase mental distress.

## Methods

### Data sources

The data utilized for this study came from the 2016 12th “Korea Youth Risk Behavior Web-based Survey (KYRBS)” [44]. The KYRBS is an annual anonymous online self-reported cross-sectional survey on various health behaviours that uses a stratified cluster sampling procedure to source middle and high school students that are representative of the adolescent school population in Korea [44], more details under [44]. The online survey was administered during class after survey instructions had been given and written informed consent had been obtained [44]. In 2016, the survey included a total of 798 schools, and a total of 65,528 respondents participated, resulting in a response rate of 96.4% [44].

### Measures

Three assessment measures of psychological well-being (self-rated health, happiness, and sleep satisfaction) and two questions on mental distress (perceived stress and depression symptoms) were used in this study.

*Self*-*rated health* was assessed with the question: “How healthy do you usually feel?” (Response option ranged from 1 = very healthy to 5 = very unhealthy) [44]. Responses were dichotomized into 1 or 2 = above average health and 3–5 = an average or below average health.

*Perceived happiness* was measured with the question: “How happy do you usually feel?” (Response options: (1) very happy, (2) happy, (3) average, (4) unhappy, or (5) very unhappy) [44]. Responses were dichotomized into 1–2 = above average happiness and 3–5 = average or below average happiness.

*Sleep satisfaction* was assessed with the question, “In the past 7 days, did you get adequate sleep to overcome fatigue?” (Response options ranged from 1 = Sufficient to 5 = Not sufficient at all) [44]. Responses were dichotomized into 1–2 = above average sufficient sleep and 3–5 = average or below average sufficient sleep.

*Perceived stress* was assessed with the question, “To what degree are you usually stressed?” (Response options arranged from 1 = very much to 5 = not at all) [44]. Responses were dichotomized into 1–2 = above average stress and 3–5 = average or below average stress.

*Depression symptoms* were assessed with the question, “Have you experienced sadness or despair to the degree that you stopped your daily routine for the recent 12 months?” (Response option, “Yes” or “No”) [44].

#### Dietary behaviours

To evaluate dietary behaviours, the regularity of breakfast meal time consumed over the past 7 days was surveyed with eight scales from 0 to 7 days. For food groups consumed over the past 7 days, the participants were asked the frequency of seven food groups, such as (1) soft drinks, (2) highly caffeinated drinks, (3) sweetened drinks, (4) fast food foods (such as pizza, hamburgers, or chicken), (5) fruits (not fruit juices), (6) vegetable dishes (excluding Kimchi), and (7) milk consumption during the past 7 days and the responses were from 1 = none, 2 = 1–2 times/week, 3 = 3–4 times/week, 4 = 5–6 times/week, 5 = once/day, 6 = twice/day, and 7 = 3 times or more/day [44].

#### Control variables

*Sociodemographic variables* included gender, age, geolocality (rural area, small or large city), maternal and paternal educational level, perceived socioeconomic status (SES), types of school (Boys only, girls only and mixed), school level (middle school and high school) [44].

*The Body Mass Index (BMI)* of students was calculated by dividing their self-reported weight in kilogrammes by their height in meters squared (kg/m^2^). According to age and gender, the students were categorized into “underweight (< 5th percentile), normal weight (5th ≤ BMI < 85th percentile), overweight (85th ≤ BMI < 95th percentile), and obese (≥ 95th percentile)”, following the BMI cut-off criteria set for Korean children by the 2007 Korean Growth Charts [45].

*Physical activity* was assessed in terms of the frequency of physical activity of ≥ 60 min per day during the past 7 days [44]. Responses were categorised into 1 = no days, 2 = 1–2 days, and 3 = 3–7 days.

*Lifetime alcohol and tobacco use* was measured with the questions, “Have you ever used alcohol?” and “Have you ever used tobacco?” (Response option, “Yes”, “No”) [44].

### Data analysis

Descriptive statistics were used to present the proportion or mean of general subject characteristics and outcome variables. Logistic regression tests were performed to estimate adjusted odds ratios (ORs) and 95% confidence intervals (CIs) after adjustment for selected covariates. Logistic regression analyses were conducted to calculate the association between the adolescents’ well-being and mental distress variables as the main outcome variables and dietary behaviour variables after adjustment for covariates selected from bivariate association analysis with outcome variables. All analyses conducted took the sampling design parameters, weighting, clustering, and stratification of the study survey into account. All values were weighted according to the participant’s probability of being chosen by sex-, grade-, and school type-specific distributions for the study region [46]. The “finite population correction (fpc) factor was used to avoid the overestimation, when developing variance estimates for population parameters” [47]. All statistical analyses was done by SAS 9.3 (SAS Institute, Cary, NC).

## Results

### Sample characteristics

The sample included 65,528 school-going adolescents (Mean age = 15.1 years, SE = 0.02; age range 12–18 years) from Korea. More than half of the sample (52.2%) were male, attended high school (54.6%), and a mixed school (62.0%). More than one-third (37.2%) of the students perceived to have a high or high-middle socioeconomic status, 63.4 and 56.0% had a father and had a mother, respectively, with college or higher education. Overall, 17.3% of the students were overweight or obese, 31.3% engaged in 60 min or more physical activity 3–4 times a week, 14.8% ever smoked and 38.8% ever drank alcohol (see Table 1).

**Table 1:** General characteristics of study participants

	Unweighted frequency	Weighted %
Sex		
Boys	33,803	52.2
Girls	31,725	47.8
Age (years), mean (sd)	65,212	15.1 (0.02)
BMI		
Thinness (< 5th percentile)	3586	5.7
Normal weight (5th ≤ BMI < 85th percentile)	48,979	77.0
Overweight (85th ≤ BMI < 95th percentile)	2994	4.5
Obesity (≥ 95th percentile)	8182	12.8
School		
High school	33,309	54.6
Middle school	32,219	45.4
Types of school		
Mixed	41,445	62.0
Boys only	12,032	19.3
Girls only	12,051	18.7
Paternal education level		
High school or less	19,610	36.6
College or higher	31,977	63.4
Maternal education level		
High school or less	23,497	44.0
College or higher	28,860	56.0
Perceived socio-economic status		
High/high-middle	24,244	37.2
Middle	31,056	47.3
Low-middle/Low	10,228	15.6
Place of residence		
Rural area	4856	5.8
Large city	29,046	43.3
Medium-sized city	31,626	50.8
Physical activity (≥ 60 min)		
No	23,817	36.8
1–2/week	20,859	32.0
3+/week	20,852	31.3
Ever smoking in lifetime (yes)	9511	14.8
Ever alcohol drinking in lifetime (yes)	24,804	38.8

All values are presented as weighted Mean (SD) or weighted % as appropriate

### Prevalence of well-being and mental distress indicators

Regarding well-being indicators, 26.5% of the students perceived themselves to be “very healthy”, 28.1% as “very happy” and 25.8% had sufficient or quite sufficient sleep satisfaction. In terms of mental distress, 37.3% of students reported somewhat or very much “perceived stress”, while 25.5% reported depression symptoms (see Table 2).

**Table 2:** Prevalence of mental health among adolescents

	Unweighted Frequency	Weighted %
1. Well-being outcomes		
Perceived health		
Very healthy	17,586	26.5
Healthy	29,647	45.3
Fair	14,223	21.9
Poor	3846	6.0
Very poor	226	0.4
Perceived happiness		
Very happy	18,992	28.1
Happy	24,964	38.5
Fair	16,743	25.8
Unhappy	4102	6.4
Very unhappy	727	1.1
Sleep satisfaction (Fatigue recovery from sleep)		
Quite sufficient	5413	7.8
Sufficient	12,081	18.0
So So	20,705	31.7
Not sufficient	18,296	28.4
Not sufficient at all	9033	14.1
2. Mental distress outcomes		
Perceived stress		
Very much	6513	10.0
Somewhat	17,833	27.3
Average	28,021	42.9
Not so much	10,772	16.2
Not at all	2389	3.6
Signs and symptoms of depression during the last year		
No	48,993	74.5
Yes	16,535	25.5

All values are presented as weighted %

### Associations between dietary behaviours with well-being and mental distress indicators

Tables 3 and 4 describe the bivariate associations with well-being and mental distress indicators, and Table 5 the adjusted analysis with well-being and mental distress indicators. In logistic regression analysis, adjusted for potential confounders, positive dietary behaviours (fruit and vegetable consumption, daily breakfast, milk consumption) were positively and unhealthy dietary behaviours (intake of caffeine, soft drinks, sweet drinks and fast food) were negatively associated with happiness or sleep satisfaction or self-reported health. Positive dietary behaviours (fruit and vegetable consumption, having daily breakfast, and milk consumption) were negatively associated with perceived stress and depression symptoms. Unhealthy dietary behaviours (fast food, caffeine, sweetened drinks and soft drinks consumption) were positively associated with perceived stress and depression symptoms (see Tables 3, 4, 5).

**Table 3:** Association between covariates and mental health among adolescents

	Well-being outcomes									Mental distress outcomes					
	Perceived health			Perceived happiness			Sleep satisfaction			Perceived stress			Depression		
	Bad	Good	p-value	Unhappy	Happy	p-value	Insufficient	Sufficient	p-value	Less	Much	p-value	No	Yes	p-value
Sex (boys)	43.2	55.7	< .0001	47.2	54.7	< .0001	47.7	64.8	< .0001	57.9	42.5	< .0001	55.4	42.7	< .0001
Age (years), mean (SD)	15.4 (0.02)	15.0 (0.02)	< .0001	15.4 (0.02)	15.0 (0.02)	< .0001	15.3 (0.02)	15.0 (0.03)	< .0001	15.0 (0.02)	15.3 (0.02)	< .0001	15.0 (0.02)	15.3 (0.02)	< .0001
BMI															
Normal weight	71.4	79.2	< .0001	76.3	77.4	0.008	77.3	76.2	0.0239	77.8	75.6	< .0001	77.0	77.1	0.3670
Thinness	7.3	5.1		5.8	5.6		5.6	6.0		5.8	5.5		5.8	5.5	
Overweight/obesity	21.3	15.7		18.0	17.0		17.1	17.9		16.4	18.8		17.2	17.5	
School level															
High school	62.3	51.6	< .0001	62.4	50.7	< .0001	60.0	39.2	< .0001	51.9	59.2	< .0001	52.9	59.5	< .0001
Middle school	37.7	48.4		37.6	49.3		40.0	60.8		48.1	40.8		47.1	40.5	
Types of school															
Mixed	60.8	62.5	< .0001	61.1	62.5	< .0001	60.6	66.1	< .0001	62.6	61.0	< .0001	61.8	62.6	< .0001
Boys only	16.8	20.3		18.0	19.9		18.5	21.4		21.3	15.9		20.7	15.2	
Girls only	22.4	17.2		21.0	17.6		20.9	12.5		16.0	23.2		17.5	22.1	
Paternal education level															
High school or less	39.8	35.3	< .0001	39.4	35.2	< .0001	37.4	34.1	< .0001	35.7	37.9	< .0001	36.4	37.1	0.1642
College or higher	60.2	64.7		60.6	64.8		62.6	65.9		64.3	62.1		63.6	62.9	
Maternal education level															
High school or less	47.9	42.5	0.0009	47.4	42.4	< .0001	45.3	40.3	< .0001	42.9	45.8	< .0001	44.0	44.2	0.7602
College or higher	52.1	57.5		52.6	57.6		54.7	59.7		57.1	54.2		56.0	55.8	
Socio-economic status															
High/upper middle	27.3	41.0	< .0001	26.4	42.6	< .0001	34.6	44.5	< .0001	39.1	33.8	< .0001	38.0	34.6	< .0001
Middle	50.1	46.1		50.4	45.7		48.5	43.7		48.2	45.7		48.1	44.7	
Lower middle/Low	22.6	12.8		23.2	11.7		16.9	11.8		12.7	20.5		13.8	20.8	
Place of residence															
Rural area	5.4	6.0	0.0016	5.6	6.0	0.006	5.7	6.3	0.2566	5.7	6.1	0.1621	38.0	34.6	< .0001
Large city	42.0	43.8		42.2	43.9		43.3	43.3		43.8	42.6		48.1	44.7	
Medium-sized city	52.6	50.1		52.2	50.1		51.0	50.4		50.5	51.3		13.8	20.8	
Physical activity (≥ 60 min)															
No	42.9	34.3	< .0001	41.0	34.7	< .0001	37.6	34.3	< .0001	35.8	38.4	< .0001	37.2	35.6	0.0011
1–2/week	34.6	30.9		32.7	31.6		32.8	29.6		31.2	33.3		31.6	33.1	
3+/week	22.5	34.7		26.4	33.7		29.6	36.0		33.1	28.3		31.3	31.3	
Ever smoking (yes)	15.7	14.5	0.0013	17.7	13.4	< .0001	15.9	11.9	< .0001	13.9	16.4	< .0001	12.9	20.4	< .0001
Ever alcohol drinking (yes)	42.0	37.5	< .0001	44.4	36.0	< .0001	41.7	30.4	< .0001	36.2	43.1	< .0001	35.5	48.3	< .0001

All values are presented as weighted mean ± SD or weighted % as appropriate

**Table 4:** Association between dietary behaviours and mental health among adolescents

Weighted %		Well-being outcomes									Mental distress outcomes					
		Perceived health			Perceived happiness			Sleep satisfaction			Perceived stress			Depression		
		Poor	Good	p-value	Unhappy	Happy	p-value	Insufficient	Sufficient	p-value	Less	Much	p-value	No	Yes	p-value
Breakfast																
0 day	14.9	16.8	14.1	< .0001	17.2	13.7	< .0001	15.5	13.1	< .0001	13.7	16.8	< .0001	14.3	16.7	< .0001
1 day	6.0	7.0	5.6		6.9	5.5		6.3	5.0		5.6	6.6		5.6	6.9	
2 days	7.4	8.4	7.0		8.4	6.9		7.7	6.4		6.9	8.2		6.9	8.6	
3 days	7.5	8.0	7.3		8.5	7.0		7.8	6.8		7.2	8.1		7.3	8.0	
4 days	6.5	7.3	6.2		6.6	6.5		6.8	5.7		6.4	6.7		6.3	7.1	
5 days	10.7	11.7	10.3		11.2	10.4		11.2	9.1		10.5	10.9		10.5	11.2	
6 days	8.6	8.3	8.8		8.3	8.8		8.9	7.9		8.8	8.4		8.7	8.6	
7 days	38.4	32.6	40.8		33.0	41.2		35.8	46.0		40.9	34.3		40.3	32.9	
Soft drinks																
I did not drink	24.2	24.5	24.1	< .0001	24.3	24.1	< .0001	23.8	25.2	< .0001	24.1	24.4	< .0001	24.8	22.4	< .0001
1–2 times/week	48.7	47.0	49.4		46.7	49.8		48.7	49.0		49.7	47.1		49.4	46.7	
3–4 times/week	18.9	19.1	18.7		19.3	18.6		19.1	18.3		18.8	19.0		18.4	20.3	
5–6 times/week	4.3	4.7	4.2		4.9	4.0		4.5	3.9		4.0	4.8		4.0	5.2	
Once/day	2.0	2.3	1.9		2.4	1.9		2.0	2.0		1.8	2.4		1.9	2.5	
Twice/day	0.9	1.1	0.8		1.1	0.8		1.0	0.7		0.8	1.0		0.8	1.2	
3+ times/day	0.9	1.3	0.8		1.3	0.8		1.0	0.8		0.7	1.3		0.7	1.5	
Highly caffeinated drink																
I did not drink	86.2	83.4	87.3	< .0001	83.0	87.8	< .0001	85.2	89.2	< .0001	88.4	82.5	< .0001	88.1	80.7	< .0001
1–2 times/week	9.9	11.2	9.3		11.4	9.1		10.4	8.2		8.7	11.8		8.9	12.7	
3–4 times/week	2.2	2.8	2.0		3.1	1.8		2.5	1.5		1.6	3.2		1.8	3.4	
5–6 times/week	0.8	1.0	0.7		1.1	0.6		0.8	0.6		0.6	1.0		0.6	1.4	
Once/day	0.5	0.8	0.4		0.8	0.4		0.6	0.2		0.3	0.8		0.4	1.0	
Twice/day	0.2	0.4	0.1		0.3	0.1		0.2	0.1		0.1	0.3		0.1	0.4	
3+ times/day	0.2	0.3	0.2		0.3	0.2		0.2	0.2		0.2	0.4		0.1	0.5	
Sweetened drinks																
I did not drink	15.4	15.1	15.5	< .0001	15.5	15.4	< .0001	14.4	18.2	< .0001	16.0	14.5	< .0001	16.3	12.8	< .0001
1–2 times/week	43.2	41.3	43.9		41.5	44.0		42.6	44.7		44.6	40.8		44.2	40.3	
3–4 times/week	26.4	26.4	26.5		26.6	26.4		27.0	24.7		26.1	27.1		25.8	28.5	
5–6 times/week	8.0	8.7	7.7		8.5	7.7		8.4	6.6		7.4	8.9		7.6	9.2	
Once/day	4.3	4.9	4.0		4.5	4.1		4.5	3.5		3.8	5.0		3.9	5.2	
Twice/day	1.5	1.9	1.4		1.8	1.4		1.7	1.1		1.2	2.1		1.3	2.3	
3+ times/day	1.2	1.7	1.0		1.5	1.0		1.2	1.1		0.9	1.7		1.0	1.8	
Fast foods																
I did not eat	22.8	21.9	23.2	< .0001	22.3	23.1	< .0001	21.8	25.9	< .0001	23.4	22.0	< .0001	23.7	20.3	< .0001
1–2 times/week	60.4	59.1	61.0		58.7	61.3		60.6	60.0		61.2	59.1		61.2	58.4	
3–4 times/week	13.7	15.1	13.1		14.9	13.0		14.4	11.5		12.8	15.1		12.7	16.5	
5–6 times/week	1.9	2.3	1.7		2.4	1.6		2.0	1.5		1.7	2.2		1.6	2.6	
Once/day	0.7	1.0	0.6		1.0	0.6		0.7	0.7		0.6	1.0		0.6	1.2	
Twice/day	0.2	0.3	0.2		0.3	0.2		0.2	0.2		0.2	0.3		0.2	0.4	
3+ times/day	0.2	0.3	0.2		0.4	0.2		0.3	0.2		0.2	0.4		0.1	0.6	
Fruits (excluding fruit juices)																
I did not eat	8.6	11.7	7.4	< .0001	11.8	7.0	< .0001	9.1	7.5	< .0001	7.6	10.5	< .0001	8.3	9.7	< .0001
1–2 times/week	28.7	32.1	27.4		32.3	27.0		30.0	25.1		27.7	30.4		28.3	29.9	
3–4 times/week	27.9	26.5	28.4		26.6	28.5		27.9	27.8		28.8	26.4		28.2	26.9	
5–6 times/week	11.5	10.4	12.0		10.4	12.1		11.3	12.2		11.9	11.0		11.8	10.8	
Once/day	12.6	10.8	13.4		10.6	13.6		12.2	14.0		13.1	11.8		12.8	12.2	
Twice/day	6.1	5.0	6.6		4.5	6.9		5.6	7.7		6.4	5.7		6.3	5.8	
3+ times/day	4.4	3.4	4.8		3.7	4.8		3.9	5.9		4.6	4.2		4.3	4.7	
Vegetable (excluding Kimchi)																
I did not eat	3.8	5.6	3.1	< .0001	5.1	3.1	< .0001	4.0	3.0	< .0001	3.1	5.0	< .0001	3.5	4.5	< .0001
1–2 times/week	15.5	19.4	13.9		18.5	14.0		16.5	12.7		14.7	16.8		15.0	17.0	
3–4 times/week	24.3	26.0	23.6		25.6	23.6		24.8	22.8		24.4	24.0		24.4	23.8	
5–6 times/week	14.2	13.3	14.5		13.6	14.4		14.0	14.5		14.5	13.6		14.4	13.5	
Once/day	13.0	12.0	13.4		12.5	13.3		12.9	13.4		13.4	12.4		13.0	13.0	
Twice/day	14.9	12.4	15.9		12.9	15.9		14.6	15.8		15.3	14.3		15.2	14.3	
3+ times/day	14.3	11.3	15.5		11.7	15.7		13.1	17.9		14.5	14.0		14.5	13.9	
Milk																
I did not drink	16.2	20.7	14.4	< .0001	19.7	14.4	< .0001	17.2	13.2	< .0001	14.4	19.1	< .0001	15.5	18.1	< .0001
1–2 times/week	22.6	25.3	21.5		24.4	21.6		23.8	19.2		21.9	23.7		22.2	23.7	
3–4 times/week	20.2	19.8	20.3		19.8	20.4		20.3	19.8		20.5	19.7		20.2	20.1	
5–6 times/week	14.3	13.1	14.7		13.4	14.7		14.0	15.1		14.8	13.4		14.6	13.2	
Once/day	16.0	12.9	17.2		13.7	17.1		15.3	18.1		16.9	14.4		16.5	14.7	
Twice/day	6.2	4.8	6.7		5.1	6.7		5.6	7.8		6.6	5.5		6.3	5.9	
3+ times/day	4.6	3.3	5.2		3.8	5.0		3.9	6.8		4.9	4.2		4.7	4.4	

All values are presented as weighted %

**Table 5:** Adjusted odds ratios of well-being and mental distress indicators in relation to dietary behaviours among adolescents

	Well-being outcomes						Mental distress outcomes			
	Perceived health (healthy)		Perceived happiness (happy)		Sleep satisfaction (sufficient)		Perceived stress (much)		Depression (yes)	
	aOR^1)^	(95% CI)	aOR^1)^	(95% CI)	aOR^2)^	(95% CI)	aOR^2)^	(95% CI)	aOR^3)^	(95% CI)
Dietary behaviors										
Breakfast										
0 day	1.00		1.00		1.00		1.00		1.00	
1 day	0.95	(0.85–1.05)	1.01	(0.92–1.11)	0.96	(0.85–1.09)	0.91	(0.83–1.00)	0.97	(0.89–1.06)
2 days	1.04	(0.95–1.14)	1.06	(0.97–1.15)	0.99	(0.89–1.11)	0.95	(0.87–1.04)	1.02	(0.94–1.10)
3 days	1.06	(0.97–1.17)	1.02	(0.94–1.11)	*1.12*	*(1.01–1.25)*	*0.91*	*(0.84–0.99)*	*0.88*	*(0.82–0.96)*
4 days	0.98	(0.89–1.08)	*1.22*	*(1.11–1.34)*	0.99	(0.88–1.11)	*0.83*	*(0.76–0.92)*	0.94	(0.87–1.02)
5 days	1.01	(0.94–1.10)	*1.16*	*(1.07–1.25)*	0.99	(0.91–1.09)	*0.85*	*(0.79–0.91)*	*0.89*	*(0.83–0.96)*
6 days	*1.22*	*(1.12–1.34)*	*1.30*	*(1.19–1.42)*	*1.13*	*(1.03–1.23)*	*0.76*	*(0.70–0.82)*	*0.86*	*(0.79–0.93)*
7 days	*1.34*	*(1.25–1.43)*	*1.42*	*(1.34–1.51)*	*1.45*	*(1.35–1.56)*	*0.74*	*(0.70–0.78)*	*0.76*	*(0.72–0.81)*
Soft drinks										
I did not drink	1.00		1.00		1.00		1.00		1.00	
1–2 times/week	1.04	(0.99–1.09)	*1.08*	*(1.03–1.13)*	*0.90*	*(0.86–0.96)*	0.97	(0.93–1.02)	*1.05*	*(1.00–1.09)*
3–4 times/week	*0.90*	*(0.84–0.96)*	0.95	(0.89–1.01)	*0.77*	*(0.72–0.82)*	*1.07*	*(1.01–1.14)*	*1.24*	*(1.17–1.31)*
5–6 times/week	*0.83*	*(0.74–0.92)*	*0.82*	*(0.74–0.91)*	*0.70*	*(0.62–0.80)*	*1.39*	*(1.25–1.54)*	*1.44*	*(1.31–1.58)*
Once/day	*0.73*	*(0.63–0.84)*	*0.76*	*(0.66–0.88)*	*0.77*	*(0.65–0.91)*	*1.47*	*(1.28–1.70)*	*1.57*	*(1.38–1.79)*
Twice/day	*0.63*	*(0.50–0.79)*	*0.77*	*(0.62–0.94)*	*0.58*	*(0.44–0.77)*	*1.41*	*(1.12–1.78)*	*1.59*	*(1.34–1.89)*
3+ times/day	*0.63*	*(0.50–0.78)*	*0.67*	*(0.53–0.84)*	0.80	(0.63–1.01)	*1.75*	*(1.41–2.18)*	*2.07*	*(1.75–2.44)*
Highly caffeinated drink										
I did not drink	1.00		1.00		1.00		1.00		1.00	
1–2 times/week	*0.77*	*(0.72–0.83)*	*0.73*	*(0.69–0.78)*	*0.68*	*(0.63–0.73)*	*1.50*	*(1.42–1.60)*	*1.50*	*(1.42–1.59)*
3–4 times/week	*0.65*	*(0.57–0.74)*	*0.55*	*(0.49–0.62)*	*0.56*	*(0.48–0.66)*	*2.22*	*(1.96–2.52)*	*1.91*	*(1.71–2.13)*
5–6 times/week	*0.58*	*(0.46–0.73)*	*0.55*	*(0.44–0.68)*	*0.70*	*(0.53–0.92)*	*1.96*	*(1.58–2.44)*	*2.66*	*(2.19–3.23)*
Once/day	*0.44*	*(0.33–0.58)*	*0.43*	*(0.34–0.55)*	*0.40*	*(0.27–0.58)*	*3.43*	*(2.67–4.41)*	*2.62*	*(2.15–3.20)*
Twice/day	*0.30*	*(0.19–0.45)*	*0.42*	*(0.26–0.69)*	*0.49*	*(0.26–0.96)*	*3.49*	*(2.28–5.34)*	*3.57*	*(2.38–5.34)*
3+ times/day	*0.39*	*(0.25–0.62)*	*0.43*	*(0.28–0.68)*	*0.77*	*(0.45–1.32)*	*3.01*	*(1.85–4.89)*	*3.25*	*(2.24–4.71)*
Sweetened drinks										
I did not drink	1.00		1.00		1.00		1.00		1.00	
1–2 times/week	1.01	(0.95–1.07)	*1.06*	*(1.00–1.12)*	*0.87*	*(0.82–0.93)*	0.99	(0.94–1.05)	*1.12*	*(1.06–1.18)*
3–4 times/week	*0.92*	*(0.86–0.99)*	0.99	(0.93–1.06)	*0.77*	*(0.71–0.83)*	*1.14*	*(1.07–1.21)*	*1.34*	*(1.26–1.41)*
5–6 times/week	*0.80*	*(0.73–0.87)*	0.95	(0.87–1.03)	*0.63*	*(0.57–0.71)*	*1.30*	*(1.21–1.41)*	*1.45*	*(1.35–1.57)*
Once/day	*0.77*	*(0.69–0.86)*	0.94	(0.84–1.05)	*0.66*	*(0.59–0.75)*	*1.47*	*(1.33–1.62)*	*1.58*	*(1.44–1.73)*
Twice/day	*0.65*	*(0.54–0.78)*	*0.81*	*(0.69–0.94)*	*0.57*	*(0.47–0.69)*	*1.82*	*(1.55–2.14)*	*2.04*	*(1.76–2.37)*
3+ times/day	*0.58*	*(0.48–0.70)*	*0.68*	*(0.57–0.82)*	*0.82*	*(0.66–1.01)*	*2.08*	*(1.73–2.50)*	*1.97*	*(1.67–2.32)*
Fast foods										
I did not eat	1.00		1.00		1.00		1.00		1.00	
1–2 times/week	0.97	(0.92–1.02)	*1.05*	*(1.01–1.11)*	*0.85*	*(0.81–0.90)*	1.01	(0.96–1.05)	*1.08*	*(1.04–1.13)*
3–4 times/week	*0.80*	*(0.75–0.86)*	*0.89*	*(0.83–0.95)*	*0.66*	*(0.62–0.72)*	*1.24*	*(1.16–1.32)*	*1.43*	*(1.35–1.52)*
5–6 times/week	*0.69*	*(0.59–0.81)*	*0.71*	*(0.61–0.82)*	*0.70*	*(0.59–0.84)*	*1.49*	*(1.28–1.72)*	*1.80*	*(1.58–2.05)*
Once/day	*0.50*	*(0.40–0.63)*	*0.52*	*(0.42–0.66)*	0.78	(0.58–1.04)	*2.03*	*(1.63–2.54)*	*2.30*	*(1.90–2.78)*
Twice/day	*0.41*	*(0.25–0.69)*	*0.50*	*(0.31–0.82)*	0.58	(0.33–1.02)	*2.14*	*(1.35–3.39)*	*2.36*	*(1.66–3.37)*
3+ times/day	1.32	(0.67–2.59)	0.73	(0.42–1.25)	0.61	(0.32–1.19)	*2.09*	*(1.24–3.52)*	*3.57*	*(2.62–4.87)*
Fruits (excluding fruit juices)										
I did not eat	1.00		1.00		1.00		1.00		1.00	
1–2 times/week	*1.32*	*(1.21–1.43)*	*1.45*	*(1.34–1.57)*	1.08	(0.98–1.18)	*0.77*	*(0.72–0.83)*	*0.88*	*(0.83–0.94)*
3–4 times/week	*1.58*	*(1.46–1.72)*	*1.76*	*(1.62–1.90)*	*1.23*	*(1.12–1.35)*	*0.67*	*(0.62–0.72)*	*0.83*	*(0.77–0.88)*
5–6 times/week	*1.61*	*(1.46–1.77)*	*1.77*	*(1.62–1.94)*	*1.29*	*(1.17–1.42)*	*0.68*	*(0.63–0.74)*	*0.83*	*(0.77–0.90)*
Once/day	*1.80*	*(1.64–1.98)*	*2.04*	*(1.86–2.23)*	*1.42*	*(1.29–1.58)*	*0.66*	*(0.61–0.71)*	*0.86*	*(0.79–0.92)*
Twice/day	*1.72*	*(1.54–1.93)*	*2.18*	*(1.95–2.44)*	*1.56*	*(1.39–1.75)*	*0.69*	*(0.62–0.76)*	*0.86*	*(0.78–0.94)*
3+ times/day	1.81	(1.58–2.07)	1.89	(1.67–2.14)	1.68	(1.49–1.90)	0.70	(0.63–0.78)	1.05	(0.95–1.17)
Vegetable (excluding Kimchi)										
I did not eat	1.00		1.00		1.00		1.00		1.00	
1–2 times/week	*1.35*	*(1.21–1.51)*	*1.26*	*(1.12–1.40)*	1.01	(0.88–1.15)	*0.69*	*(0.62–0.77)*	*0.90*	*(0.82–1.00)*
3–4 times/week	*1.68*	*(1.51–1.87)*	*1.49*	*(1.34–1.65)*	*1.17*	*(1.03–1.32)*	*0.63*	*(0.57–0.70)*	*0.79*	*(0.72–0.87)*
5–6 times/week	*1.90*	*(1.69–2.14)*	*1.61*	*(1.44–1.80)*	*1.28*	*(1.12–1.46)*	*0.62*	*(0.56–0.70)*	*0.80*	*(0.72–0.88)*
Once/day	*1.93*	*(1.73–2.16)*	*1.61*	*(1.44–1.81)*	*1.27*	*(1.11–1.45)*	*0.62*	*(0.55–0.69)*	*0.84*	*(0.76–0.93)*
Twice/day	*2.22*	*(1.97–2.49)*	*1.87*	*(1.67–2.10)*	*1.35*	*(1.18–1.53)*	*0.61*	*(0.55–0.68)*	*0.78*	*(0.70–0.86)*
3+ times/day	*2.21*	*(1.97–2.48)*	*1.96*	*(1.75–2.19)*	*1.56*	*(1.37–1.77)*	*0.66*	*(0.59–0.74)*	*0.83*	*(0.75–0.92)*
Milk										
I did not drink	1.00		1.00		1.00		1.00		1.00	
1–2 times/week	*1.15*	*(1.08–1.24)*	*1.15*	*(1.08–1.22)*	1.00	(0.93–1.08)	*0.84*	*(0.79–0.89)*	*0.93*	*(0.88–0.98)*
3–4 times/week	*1.28*	*(1.20–1.36)*	*1.28*	*(1.20–1.36)*	*1.09*	*(1.01–1.18)*	*0.82*	*(0.77–0.87)*	*0.93*	*(0.88–0.99)*
5–6 times/week	*1.33*	*(1.23–1.44)*	*1.32*	*(1.23–1.41)*	1.07	(0.98–1.16)	*0.80*	*(0.75–0.86)*	*0.89*	*(0.84–0.95)*
Once/day	*1.50*	*(1.39–1.61)*	*1.41*	*(1.32–1.51)*	*1.18*	*(1.09–1.28)*	*0.77*	*(0.72–0.82)*	*0.90*	*(0.85–0.96)*
Twice/day	*1.48*	*(1.33–1.64)*	*1.36*	*(1.22–1.51)*	*1.21*	*(1.10–1.34)*	*0.83*	*(0.76–0.91)*	1.02	(0.94–1.11)
3+ times/day	*1.54*	*(1.36–1.74)*	*1.37*	*(1.22–1.53)*	*1.46*	*(1.31–1.63)*	*0.90*	*(0.82–1.00)*	1.06	(0.96–1.17)

## Discussion

This study found in agreement with previous studies [1–3] that a dose–response relationship between healthy dietary behaviours (regular fruit, vegetable, breakfast, and milk consumption) and well-being outcomes (perceived health, happiness and sleep satisfaction). In particular, the linear association with positive perceived health and happiness were stronger in fruit and vegetable consumption. A study among ASEAN university students showed a significant association but no dose–response relationship between fruits and vegetable consumption and positive self-rated health status [6]. Hoefelmann et al. [48] also found that higher fruit and vegetables consumption was associated with better sleep quality among Brazilian workers. Reasons for this finding are not clear and need further investigations.

Recent meta-analyses confirmed an inverse association of healthy dietary patterns [49, 50] with poor mental health outcomes, like depression in adults. However, the findings in adolescents remained inconsistent. In agreement with previous studies [1–3, 9–25], this study found that healthy dietary behaviours (regular fruit, vegetable, breakfast, and milk consumption) were negatively associated with perceived stress and depression symptoms, despite no linear associations of consumption of fruit, vegetable, and milk. A population-based study among Swiss people aged 15+ years showed those fulfilling the 5-a-day fruit and vegetable consumption had lower odds of being highly or moderately distressed than individuals consuming less fruit and vegetables (OR  =  0.82 for moderate distress, and OR  =  0.55, for high distress compared to low distress) [31]. It is possible that due to the consumption of fruits and vegetables, being rich in antioxidants, folic acid and anti-inflammatory components, human optimism or happiness is enhanced [28] and the development of negative mood or depression symptoms decreased [29].

In agreement with previous studies [8, 24–31, 35] unhealthy dietary behaviours (consumption of soft drinks, caffeine, fast food, sweets and snacks, and skipping breakfast) were associated with low self-rated health, unhappiness, and low sleep satisfaction. Although the association became weaker at three or more times consumption of fast foods, increased unhealthy dietary behaviours were inversely associated with positive well-being outcomes, in particular, perceived health and happiness. On the other hand, a dose–response relationship between unhealthy dietary behaviours, such as consumption of soft drinks, highly caffeinated drinks, sweetened drinks, and fast food, and inversely, frequency of breakfast consumption as a health dietary behaviour with depression was observed in this study. These findings are consistent with a prospective Australian adolescents study [51] and a prospective cohort study also showed a positive association of fast food and commercial baked foods with depression in adults [52]. However, in a study among university students in ASEAN countries an inverse dose–response relationship between eating breakfast and sugared coffee/tea and a positive linear association between the consumption of snacks, fast foods, soft drinks and depression symptoms [6]. Although the relationship between sugar consumption and major depression seems to have been confirmed in cross-national observations in Asian countries [53], a study among ASEAN university students has shown an inverse dose–response relationship between sugared coffee/tea consumption and depression symptoms [6]. These findings emphasize the need for further investigations.

Nevertheless, some studies have suggested that an increase in carbohydrate-dense but nutrient-poor foods, such as fast food, sweets and snacks, may be used by individuals to cope with negative mood and elevate mood by increasing brain serotonin levels [42]. Several other studies among adolescents [54] and young adults [55] also found an association between caffeine consumption and low sleep satisfaction or poor sleep quality. A study among adolescents in Germany suggested that later bed and rise times were associated with increased consumption of caffeinated drinks and fast food [56]. The biological mechanism to explain this includes that caffeine increases alertness and increased energy as a function of its interactions with adenosine receptors in the brain [57]. However, caffeine use seems to only reduce sleep quality in individuals that are sensitive to the adenosine effects of caffeine [58]. In addition, the German study reported reduced consumption of dairy products was also associated with later bed and rise times [56]. Our study findings supported this study by showing that frequent milk consumption (once per day or more) was associated with sufficient sleep satisfaction. Further, as the practice of skipping breakfast may increase poor sleep quality [30], our study also showed a positive association between regular breakfast consumption and sleep satisfaction. In terms of fast foods, less frequent consumption of fast foods (less than once per day) showed an inverse association, but among those having once per day or more fast foods the association disappeared. This study may lead to a need for a prospective study to examine the causality, since strong relationships with a dose–response relationship between healthy dietary behaviours and well-being parameters and between unhealthy dietary behaviours and mental distress were found.

### Study limitations

The cross-sectional design does not explain if positive well-being promotes a healthier dietary behaviour or healthier dietary patterns lead to more positive well-being. Some of the concepts assessed in this study used single item measures such as depression symptoms, happiness and perceived stress, and future studies should include multiple item measures to assess key concepts. Despite the limitations, the inclusion of data from 65,528 adolescents from a nationally representative sample in South Korea supports the external validity of the study results.

## Conclusions

In a large nationally representative sample of adolescent in Korea, strong cross-sectional evidence was found that increased unhealthier dietary behaviour was associated with higher mental distress, while healthier dietary behaviour showed a dose–response relationship with higher psychological well-being. It remains unclear, if a healthier dietary behaviour is the cause or the sequela of a more positive well-being.

## Abbreviations

BMI: Body Mass Index
KYRBS: Korea Youth Risk Behavior Web-based Survey
